# Comparative Analysis of the Volatile Fraction of Fruit Juice from Different *Citrus* Species

**DOI:** 10.1371/journal.pone.0022016

**Published:** 2011-07-19

**Authors:** M. Carmen González-Mas, José Luis Rambla, M. Carmen Alamar, Abelardo Gutiérrez, Antonio Granell

**Affiliations:** 1 Centro de Citricultura, Instituto Valenciano de Investigaciones Agrarias (IVIA), Moncada, Valencia, Spain; 2 Instituto de Biología Molecular y Celular de Plantas (IBMCP), CSIC - Universidad Politécnica de Valencia, Valencia, Spain; 3 Centro de AgroIngeniería, Instituto Valenciano de Investigaciones Agrarias (IVIA), Moncada, Valencia, Spain; Università di Napoli Federico II, Italy

## Abstract

The volatile composition of fruit from four *Citrus* varieties (Powell Navel orange, Clemenules mandarine, and Fortune mandarine and Chandler pummelo) covering four different species has been studied. Over one hundred compounds were profiled after HS-SPME-GC-MS analysis, including 27 esters, 23 aldehydes, 21 alcohols, 13 monoterpene hydrocarbons, 10 ketones, 5 sesquiterpene hydrocarbons, 4 monoterpene cyclic ethers, 4 furans, and 2 aromatic hydrocarbons, which were all confirmed with standards. The differences in the volatile profile among juices of these varieties were essentially quantitative and only a few compounds were found exclusively in a single variety, mainly in Chandler. The volatile profile however was able to differentiate all four varieties and revealed complex interactions between them including the participation in the same biosynthetic pathway. Some compounds (6 esters, 2 ketones, 1 furan and 2 aromatic hydrocarbons) had never been reported earlier in *Citrus* juices. This volatile profiling platform for *Citrus* juice by HS-SPME-GC-MS and the interrelationship detected among the volatiles can be used as a roadmap for future breeding or biotechnological applications.

## Introduction

Developing powerful platforms for volatile analysis is a prerequisite for further insights into the volatiles biosynthetic pathways and also in the identification of the genetic and environmental effects in volatile production [1], [2]. This information is relevant in the frame of current breeding programs in *Citrus* which are directed to respond to the market demand for quality fruits and are also important for the biotechnology of fruit and fruit derived product. One of the main characteristics of *Citrus* fruit quality is defined by the aroma of fruit juice. The aroma of a fresh juice is the product of a complex combination of several odour components that include esters, aldehydes, alcohols, ketones and hydrocarbons, which are collectively defined as volatile organic compounds or VOCs [3]–[6]. Headspace extraction coupled to GC-MS is at present the method of choice for most of the volatile analysis in food/flavour chemistry [7], [8] and particularly in *Citrus* [9]–[17], having displaced former methods that involved complex sample preparation and large amounts of solvents [18]–[20]. Some studies on the compositional analysis of *Citrus* juice aroma have been described which used dynamic and static headspace extraction [21], [22]. Different types of fibers have been used for *Citrus* juice analysis by HS-SPME [10], [13], [14], [23] but the one with three components: DVB/CAR/PDMS (divinylbenzene/ carboxen/polydimethylsiloxane) is the most widely used, because of its ability to extract a larger number of VOCs than other fibers [15], [17], [24], [25].

So far, almost all the studies on the aroma of *Citrus* juices had been conducted on orange juice, normally using one or at most two varieties. The fragmented information available together with the different techniques and fibers used, complicates the comparison of VOCs profiles between different *Citrus* varieties present in the literature [5], [10], [15], [16], [18], [19], [21], [26]–[28]. In contrast to oranges, only few studies have been conducted on mandarin [10], [11] and grapefruit aroma juices [4], [12], [29]. No studies have been performed for the volatiles in the juice of pummelo, and only one comparative study has been reported comparing mandarin and orange juices [3]. In this paper we describe the optimization of a VOCs capture/profiling method for *Citrus* and the characterization of the volatile profile for the juice of four *Citrus* varieties: Powell Navel summer orange, Clemenules clementine mandarine, and Fortune mandarine and Chandler pummelo hybrids. All four varieties are used as parentals in order to obtain new hybrids in breeding programs and at the same time they are themselves important varieties for fresh market in the world [30]. This is the first time that different varieties corresponding to different species are analysed in parallel using the same analytical technique and therefore enable us to describe both the volatile fraction in the juice and the variability in the volatile profile between the materials analysed. It is also the first time that the volatiles in the juice of pummelo are described.

## Materials and Methods

### *Citrus* juice

Mature fruits at optimal ripening stage [31], were collected in 2007 from trees of Powell Navel Late sweet orange (*Citrus sinensis* (L.) Osb.), Clemenules (*Citrus clementine* Hort. ex Tan.), and two *Citrus* hybrids: Fortune (*C. clementine* x *C. tangerine*) and Chandler pummelo (*C. grandis* x *C. grandis*) varieties. All trees were grown in the same orchard and subjected to homogeneous cultural conditions, in order to reduce environmental effects on the volatile profile. The experimental orchard is located at the Experimental Station of *Instituto Valenciano de Investigaciones Agrarias*, Moncada, Valencia, Spain, under a mediterranean climate (averages rainfall of 515.8 mm and temperature of 15.2°C for 2007). In all cases, three biological replicate samples for each variety were obtained, each one representing at least four different fruits each. Fruit juice was obtained using a hand extractor, in order to avoid squeezing of the flavedo and to prevent contamination of the juice with peel components. After that, 10 mL aliquots of each sample were placed in 22 mL crimp cap headspace vials and kept frozen at −20°C until analyzed. Two aliquots of 10 mL corresponding to technical replicates of each sample were analyzed. The total number of analysis was 24 (3 biological samples x 2 technical replicates for the 4 varieties).

### HS-SPME extraction conditions

Right before analysis, samples were thawed at 20°C for ten minutes and then were subjected to headspace solid phase micro-extraction (HS-SPME). Extraction was carried out using 10 mL of sample into a 22 mL crimp cap headspace vial. A 50/30 µm DVB/CAR/PDMS (Supelco, Bellefonte, PA, USA) fiber was used for all the analysis. Pre-incubation and extraction times were 10 and 20 min, respectively. A temperature of 50°C was selected for pre-incubation and extraction because it allowed the detection of a higher number of VOCs than when 30°C was used. Desorption was performed for 1 min at 250°C in splitless mode.

### Gas chromatography–mass spectrometry conditions

VOCs trapped on the fiber were analysed by GC-MS using an autosampler COMBI PAL CTC Analytics (Zwingen, Switzerland), a 6890N GC Agilent Technologies (Santa Clara, CA, USA) and a 5975B Inert XL MSD Agilent, equipped with an Agilent J&W Scientific DB-5 ms fused silica capillary column (5%-phenyl-95%-dimethylpolysiloxane as stationary phase, 60 m length, 0.25 mm i.d., and 1 µm thickness film). Oven temperature conditions were 40°C for 2 min, 5°C/min ramp until 250°C and then held isothermally at 250°C for 5 min. Helium was used as carrier gas at 1.2 mL/min constant flow. Mass/z detection was obtained by an Agilent mass spectrometer operating in the EI mode (ionization energy, 70 eV; source temperature 230°C). Data acquisition was performed in scanning mode (mass range *m/z* 35–220; seven scans per second). Chromatograms and spectra were recorded and processed using the Enhanced ChemStation software for GC-MS (Agilent).

### Compound identification

Compound identification was based both on the comparison between the MS for each putative compound with those of the NIST 2005 Mass Spectral library and also with the match to our GC retention time and Mass Spectra custom library which have been generated using commercially available compounds. Compounds used as reference were of analytical grade and purchased from Sigma-Aldrich Química (Madrid, Spain), except for 2-carene, thymol and ledene, which were obtained from Extrasynthese (Genay, France). In addition to the commercial compounds, seven esters (methyl pentanoate, ethyl pentanoate, methyl heptanoate, ethyl heptanoate, methyl octanoate, methyl nonanoate, and ethyl nonanoate) were synthesized in our laboratory by acid-catalyzed esterification from analytical grade reagents. For that, 10 µL of the corresponding acid (pentanoic acid, heptanoic acid, octanoic acid, or nonanoic acid, supplied by Sigma-Aldrich) was added to 1 mL of the corresponding alcohol (methanol, ethanol) with 10 µL of H_2_SO_4_ 96%, and incubated at 40°C overnight. After that, a small amount of sodium carbonate was added and incubated at 4°C for 24 hours, to neutralize any remaining acid. The solution was centrifuged and the supernatant used as a ≈1% standard solution of the ester in the respective alcohol. Also, 1 mL of either 100 ppb or of 1 ppm standard solutions was analyzed in the same conditions as the samples. Only those compounds/peaks confirmed by both mass spectrum and retention time in each and every chromatogram were considered. For relative quantification, the peak area was integrated from the extracted ion chromatogram corresponding to a specific ion previously selected for each compound. A mixture of extracts representing the four varieties analysed was injected regularly as part of the injection series and was used as a reference for correction for temporal variation and fiber aging. Finally, corrected results for each compound were expressed as relative ratios to the average level present in Chandler juice. When a compound was not detected in Chandler, the ratio was calculated to a variety that contained it as indicated in Table 1.

**Table 1:** Relative levels (fold changes) of VOCs detected in juices of four *Citrus* varieties.

*Code*	*Cluster*	*Volatile Organic Compound*	*Family Code/Number*	*Retention Time* (min)	*Specific Ion* (m/z)	*Chandler*	*Clemenules*	*Fortune*	*Powell*
**1**	**A1**	2-carene	*Mt hd/1*	24.05	93	1±0.25	-	traces	traces
**2**	**A1**	*(Z)*-linalool oxide^a^^,^ b	*Alc/1*	26.47	111	1±0.24	-	traces	-
**3**	**A1**	*(E)*-linalool oxide^a^^,^ b	*Alc/2*	27.00	111	1±0.22	-	-	-
**4**	**A1**	*(E,E)*-2,4-nonadienal	*Ald/1*	31.10	81	1±0.31	-	-	-
**5**	**A1**	*(Z)*-ocimene	*Mt hd/2*	24.87	93	1±0.61	-	-	-
**6**	**A1**	*p*-cymene	*Mt hd/3*	24.91	119	1±0.85	-	-	-
**7**	**A1**	β-caryophyllene	*Sqt/1*	37.87	133	1±0.57	-	-	traces
**8**	**A1**	nootkatone^c^	*Ket/1*	47.87	121	1±0.33b	-	0.02±0.01a	0.02±0.01a
**9**	**A1**	α-humulene	*Sqt/2*	38.82	80	1±0.64b	traces	0.04±0.01a	0.03±0.01a
**10**	**A1**	β-pinene	*Mt hd/4*	23.46	93	1±0.88b	0.11±0.01a	0.17±0.03a	0.21±0.12a
**11**	**A1**	1-pentanol	*Alc/3*	14.60	42	1±0.48b	0.26±0.13a	0.11±0.02a	0.20±0.11a
**12**	**A1**	1-hexanol	*Alc/4*	18.55	56	1±0.70b	0.12±0.02a	0.22±0.18a	0.87±0.32b
**13**	**A1**	methyl hexanoate	*Est/1*	20.68	74	1± 0.46c	-	0.07±0.02a	0.44±0.20b
**14**	**A1**	pseudocumene*	*Ar/1*	23.85	105	1±0.18b	0.35±0.21a	0.24±0.01a	1.11±0.43b
**15**	**A1**	ethyl hexanoate	*Est/2*	23.37	88	1±0.66b	0.17±0.06a	0.62±0.11ab	2.47±1.57c
**16**	**A2**	*(E,E)*-2,4-decadienal	*Ald/2*	34.17	81	1±0.68c	0.91±0.76bc	0.24±0.08ab	0.05±0.02a
**17**	**A2**	6-methyl-5-hepten-2-one^d^	*Ket/2*	23.03	108	1±0.40b	0.46±0.05a	0.12±0.02a	-
**18**	**A2**	octanal	*Ald/3*	23.77	57	1±0.22c	0.83±0.19b	0.39±0.05a	0.22±0.06a
**19**	**A2**	heptanal	*Ald/4*	20.00	70	1±0.26c	0.53±0.04b	0.42±0.01b	0.08±0.06a
**20**	**A2**	nonanal	*Ald/5*	27.30	57	1±0.15c	0.71±0.15b	0.60±0.06b	0.15±0.27a
**21**	**A2**	2,3-pentanedione	*Ket/3*	11.91	100	1±0.28d	0.79±0.15c	0.53±0.16b	0.07±0.04a
**22**	**A2**	*(E)*-2-octenal	*Ald/6*	25.79	70	1±0.24c	0.67±0.37b	0.51±0.15b	0.13±0.08a
**23**	**A2**	*(E)*-2-nonenal	*Ald/7*	29.21	70	1±0.28b	1.07±0.09b	0.98±0.20b	0.18±0.09a
**24**	**A2**	*(E)*-2-heptenal	*Ald/8*	22.14	83	1±0.26b	0.29±0.12a	0.23±0.05a	0.08±0.04a
**25**	**A2**	1-octen-3-one	*Ket/4*	22.82	70	1±0.46b	0.15±0.09a	0.19±0.07a	0.10±0.05a
**26**	**A2**	1-octen-3-ol	*Alc/5*	22.85	57	1±0.32b	0.11±0.02a	0.14±0.03a	0.08±0.04a
**27**	**A2**	hexanal	*Ald/9*	16.01	56	1±0.22b	0.22±0.03a	0.23±0.02a	0.14±0.07a
**28**	**A2**	pentanal	*Ald/10*	12.04	58	1±0.24b	0.19±0.02a	0.23±0.04a	0.14±0.09a
**29**	**A2**	2-pentylfuran	*Fur/1*	23.36	138	1±0.19b	0.27±0.01a	0.29±0.05a	0.14±0.12a
**30**	**A2**	1-heptanol	*Alc/6*	22.39	70	1±0.55b	0.38±0.08a	0.58±0.32a	0.31±0.05a
**31**	**A2**	α-copaene	*Sqt/3*	36.41	119	1±0.58b	0.22±0.05a	0.55±0.06a	0.35±0.02a
**32**	**A2**	valencene	*Sqt/4*	39.69	133	1±0.56b	0.15±0.02a	1.16±0.32b	0.91±0.17b
**33**	**A2**	bornyl acetate^a^	*Est/3*	33.48	121	1±0.87a	0.46±0.18a	0.73±0.13a	0.50±0.27a
**34**	**A2**	ethyl heptanoate	*Est/4*	26.82	88	1±0.67b	0.36±0.18a	1.76±0.31c	0.69±0.29ab
**35**	**A2**	propyl acetate^e^ *	*Est/5*	12.52	61	-	-	1±0.53	-
**36**	**B**	2-ethylfuran	*Fur/2*	12.17	81	1±0.21b	2.41±0.32d	1.22±0.24c	0.26±0.08a
**37**	**B**	2-methylfuran	*Fur/3*	8.84	82	1±0.25b	1.62±0.20c	1.13±0.29b	0.40±0.09a
**38**	**B**	*(E)*-2-pentenal	*Ald/11*	14.30	83	1±0.45b	4.09±0.80c	1.45±0.25b	0.41±0.07a
**39**	**B**	1-penten-3-one	*Ket/5*	11.57	55	1±0.52a	9.49±2.20c	2.45±0.59b	0.53±0.11a
**40**	**B**	ethyl propanoate	*Est/6*	12.47	57	1±1.08a	20.09±3.13b	22.36±7.40b	-
**41**	**B**	ethyl 2-methylbutanoate^f^	*Est/7*	17.87	102	-	1±0.38b	0.16±0.04a	-
**42**	**B**	3-pentanone*	*Ket/6*	11.97	57	1±0.63b	2.55±0.57c	0.59±0.19ab	0.40±0.14a
**43**	**B**	1-penten-3-ol	*Alc/7*	11.46	57	1±0.64a	2.05±1.38b	0.54±0.14a	0.71±0.37a
**44**	**B**	β-citronellala,f f	*Ald/12*	28.92	69	-	1±1.53	-	-
**45**	**B**	eucalyptol (1,8-cineole)	*Mt cyc ether/1*	25.38	154	1±0.62a	6.56±1.71c	0.30±0.06a	2.79±1.07b
**46**	**B**	decanal	*Ald/13*	30.59	57	1±0.19a	4.51±1.67b	0.78±0.22a	1.46±1.24a
**47**	**B**	*(Z)*-3-hexenal	*Ald/14*	15.92	69	1±0.89ab	1.54±0.32b	0.44±0.14a	0.81±0.54ab
**48**	**B**	*(E)*-2-hexenal	*Ald/15*	18.17	83	1±0.70a	3.92±0.81c	1.02±0.16ab	1.68±0.83b
**49**	**B**	geranylacetone^d^	*Ket/7*	37.62	43	1±0.26b	4.73±0.36d	0.58±0.14a	1.65±0.67c
**50**	**B**	β-cyclocitral^d^	*Ald/16*	31.63	137	1±0.42a	6.45±0.60b	0.78±0.13a	0.92±0.12a
**51**	**B**	β-ionone^d^	*Ket/8*	38.90	177	1±0.44a	11.34±0.76c	0.88±0.18a	1.53±0.24b
**52**	**C1**	nerol^a^	*Alc/8*	31.28	93	1±0.47ab	0.44±0.19a	1.86±2.07b	5.43±2.18c
**53**	**C1**	hexyl acetate	*Est/8*	23.86	56	1±0.86a	0.46±0.10a	0.98±0.22a	3.91±0.65b
**54**	**C1**	methyl nonanoate*	*Est/9*	30.91	74	1±0.51a	0.45±0.17a	1.02±0.11a	6.04±1.81b
**55**	**C1**	neryl acetate^a^	*Est/10*	34.97	69	1±0.55a	0.20±0.07a	1.90±0.19a	15.53±5.87b
**56**	**C1**	*(Z)*-3-hexen-1-ol	*Alc/9*	18.16	82	1±0.92a	0.50±0.11a	0.33±0.09a	4.75±1.27b
**57**	**C1**	methyl octanoate*	*Est/11*	27.74	74	1±0.49b	0.41±0.05a	0.44±0.08ab	4.60±1.39c
**58**	**C1**	geranial^a^	*Ald/17*	32.61	69	1±0.41a	0.76±0.17a	0.39±0.11a	14.62±12.11b
**59**	**C1**	geraniol	*Alc/10*	31.95	69	1±0.63a	0.78±0.12a	1.50±0.47a	10.88±6.14b
**60**	**C1**	heptyl acetate^g^ *	*Est/12*	27.27	43	-	-	-	1±0.27
**61**	**C1**	methyl decanoate*	*Est/13*	33.94	74	1±0.82a	3.78±1.48a	3.14±0.92a	72.44±42.64b
**62**	**C1**	1-nonanol	*Alc/11*	29.36	70	1±0.37a	1.38±0.57a	0.82±0.30a	12.04±10.54b
**63**	**C1**	undecanal	*Ald/18*	33.67	57	1±0.18ab	1.23±0.19b	0.76±0.09a	1.21±0.63b
**64**	**C1**	*(E)*-2-hexen-1-ol^f^	*Alc/12*	18.48	57	-	1±0.75a	-	2.72±0.80b
**65**	**C1**	1-decanol^f^	*Alc/13*	32.50	115	-	1±0.40a	0.03±0.01a	5.10±2.13b
**66**	**C1**	1-octanol	*Alc/14*	26.00	56	1±0.33a	6.38±1.83a	2.18±0.26a	59.82±50.05b
**67**	**C1**	3-carene	*Mt hd/5*	24.43	93	1±0.56a	14.10±8.38b	1.39±0.64a	78.68±4.74c
**68**	**C2**	β-citronellol^a^	*Alc/15*	31.15	81	1±0.53a	1.47±0.39a	4.53±0.45c	2.21±0.16b
**69**	**C2**	ethyl acetate	*Est/14*	9.18	61	1±0.42a	5.28±1.55b	40.71±7.12c	3.70±1.37b
**70**	**C2**	*(Z)*-carveol^a^	*Alc/16*	31.84	109	1±0.49a	4.77±1.76b	6.91±0.85c	4.85±1.98b
**71**	**C2**	*(E)*-carveol^a^	*Alc/17*	31.41	109	1±0.36a	2.91±0.19b	5.35±0.71c	2.49±0.35b
**72**	**C2**	carvone^a^	*Ket/9*	32.31	82	1±0.68a	12.09±3.13c	20.89±1.02d	5.75±0.13b
**73**	**C2**	linalool^a^	*Alc/18*	27.15	93	1±0.69a	15.73±1.35c	26.77±1.66d	10.05±0.85b
**74**	**C2**	ethanol	*Alc/19*	5.64	45	1±0.59a	10.91±2.39c	6.05±1.64b	4.95±2.33b
**75**	**C2**	acetaldehyde	*Ald/19*	4.77	43	1±0.25a	3.07±0.84b	2.75±0.22b	2.96±0.49b
**76**	**C2**	dodecanal	*Ald/20*	36.55	57	1±2.35a	77.62±21.86d	20.38±8.79b	58.44±39.38c
**77**	**C2**	3-methylfuran*	*Fur/4*	9.16	82	1±0.32a	4.25±1.60c	2.91±0.94b	2.51±0.33b
**78**	**C2**	*(E)*-limonene oxide^h^	*Mt cyc ether/2*	28.84	94	1±0.77a	7.53±2.60b	6.81±4.59b	5.98±3.14b
**79**	**C2**	*(Z)*-limonene oxide^h^	*Mt cyc ether/3*	28.77	67	1±1.15a	2.88±0.82b	3.47±0.14b	3.74±0.50b
**80**	**C3**	camphene	*Mt hd/6*	22.44	93	1±1.04a	1.37±0.17ab	1.95±0.20b	3.31±1.69c
**81**	**C3**	terpinolene	*Mt hd/7*	27.05	121	1±0.40a	3.19±0.18b	4.78±0.33c	6.11±1.01d
**82**	**C3**	limonene	*Mt hd/8*	25.11	108	1±0.25a	2.23±0.12b	2.46±0.10b	2.93±0.42c
**83**	**C3**	α-pinene	*Mt hd/9*	21.67	93	1±0.41a	6.17±0.92b	9.17±1.18b	18.56±9.66c
**84**	**C3**	myrcene	*Mt hd/10*	23.28	91	1±0.42a	2.54±0.37b	3.21±0.28b	5.58±1.78c
**85**	**C3**	α-phellandrene	*Mt hd/11*	24.29	93	1±0.41a	3.51±0.41b	5.64±0.58b	14.03±8.35c
**86**	**C3**	α-terpineol^a^	*Alc/20*	30.75	59	1±0.59a	3.78±0.25b	5.67±0.69c	6.45±3.56c
**87**	**C3**	γ-terpinene	*Mt hd/12*	26.03	93	1±0.61a	2.97±0.44b	5.16±0.85c	5.34±2.62c
**88**	**C3**	terpinen-4-ol^a^	*Alc/21*	30.38	93	1±0.77a	5.65±1.41b	15.86±2.59d	10.98±5.85c
**89**	**C3**	neral^a^	*Ald/21*	31.81	84	1±0.55a	4.28±1.43b	6.21±0.69c	7.32±1.45d
**90**	**C3**	perillaldehyde^a^	*Ald/22*	33.43	68	1±0.29a	8.81±2.43b	15.19±5.28c	25.48±2.64d
**91**	**C3**	α-terpinene	*Mt hd/13*	24.65	121	1±0.52a	3.41±1.43b	4.75±1.80c	6.19±1.61d
**92**	**C3**	ethyl nonanoate*	*Est/15*	33.05	88	1±0.91a	0.41±0.15a	5.59±1.51b	6.88±1.16c
**93**	**C3**	geranyl acetate^a^	*Est/16*	35.50	69	1±0.96a	1.19±0.34a	14.09±1.39b	28.56±12.68c
**94**	**C3**	*(Z)*-carvyl acetate^a^	*Est/17*	34.50	84	1±0.46a	0.54±0.10a	4.27±1.58b	6.92±1.84b
**95**	**C3**	*(E)*-carvyl acetate^a^	*Est/18*	35.30	84	1±1.17a	0.62±0.15a	7.36±4.56b	4.79±0.55b
**96**	**C3**	citronellyl acetate^a^	*Est/19*	34.69	95	1±0.62a	0.94±0.31a	47.41±4.01c	19.52±3.25b
**97**	**C3**	styrene*	*Ar/2*	20.01	104	1±0.30a	2.41±0.91a	2.90±0.68a	15.10±5.92b
**98**	**C3**	ethyl octanoate	*Est/20*	30.04	88	1±0.55a	1.28±0.37a	4.05±0.81b	16.63±2.00c
**99**	**C3**	nonyl acetate	*Est/21*	33.47	98	1±2.35a	4.88±6.60a	22.54±6.43a	304.38±77.26b
**100**	**C3**	ethyl butanoate	*Est/22*	15.88	88	1±2.036a	17.58±4.13b	27.11±5.07b	113.73±54.23c
**101**	**C3**	decyl acetate	*Est/23*	36.29	70	1±1.51a	48.18±18.61b	68.70±11.40c	553.34±30.06d
**102**	**C3**	α-terpinyl acetate^a^^,^ f	*Est/24*	35.10	121	-	1±0.50a	1.47±0.34b	9.61±0.72c
**103**	**C3**	linalyl acetate^a^	*Est/25*	31.82	93	1±0.47a	7.09±4.24a	7.29±0.92a	83.29±21.54b
**104**	**C3**	ethyl decanoate	*Est/26*	35.91	88	1±0.78a	5.84±1.75ab	16.43±3.04b	62.91±32.20c
**105**	**C3**	octyl acetate	*Est/27*	30.47	70	1±0.53a	18.86±8.93a	64.08±0.30b	526.00±86.22c
**106**		3-methylbutanal	*Ald/23*	10.66	58	-	traces	-	-
**107**		1,4-cineole	*Mt cyc ether/4*	24.54	111	-	traces	-	traces
**108**		β-farnesene	*Sqt/5*	37.73	120	-	-	-	traces
**109**		γ-dodecalactone*	*Ket/10*	43.71	85	traces	-	-	-

Data are normalized to the mean values in the Chandler variety, unless otherwise indicated. Mean corresponding to n = 6 values. Means followed by different letters in the same row are significantly different (p<0.05) by Duncan's text. Family Code: *Ald*: Aldehyde; *Ket*: Ketone; *Alc*: Alcohol; *Est*: Ester; *Fur*: Furane; *Mt hd*: Monoterpene hydrocarbon; *Sqt*: Sesquiterpene; *Ar*: Aromatic hydrocarbon; *Mt cyc ether*: Monoterpene cyclic ether.

a Monoterpene derived compound.

b In addition to the alcohol group, it has a tetrahydrofuran group.

c Sesquiterpene compound.

d Norcarotenoid compound.

e Data normalized to the mean abundance in Fortune variety.

f Data normalized to the mean abundance in Clemenules variety.

g Data normalized to the mean abundance in Powell variety.

h Its cyclic ether group is an epoxy group.

*Compound reported for the first time in a *Citrus* juice.

### Statistical analysis

For both Principal Component Analysis (PCA) and Hierarchical Cluster Analysis, the complete dataset including all replicates was considered. For both type of analysis, the ratio of the signal relative to that of the average in the four varieties was log 2 transformed. For PCA, the program SIMCA-P version 11 (Umetrics, Umea, Sweden) was used with the centered data. For the Hierarchical Cluster Analysis, the program Acuity 4.0 (Axon Instruments) was used, with the distance measures based on the Pearson correlation. Pearson correlation coefficients were calculated with the SPSS version 15.0 software (SPSS Inc., Chicago, USA). Data from the correlation matrix was represented as a heatmap by means of the Acuity 4.0 program.

## Results and Discussion

Table 1 lists the VOCs detected in our HS-SPME-GC-MS platform and the relative levels for the four varieties analyzed. A total of 109 compounds have been identified: 27 esters (19 aliphatic and 8 monoterpenic acetates), 23 aldehydes (18 aliphatic, 4 monoterpenic and 1 norcarotenoid), 21 alcohols (12 aliphatic and 9 monoterpenic), 13 monoterpene hydrocarbons, 10 ketones (8 aliphatic, 1 norcarotenoid and 1 monoterpenic), 5 sesquiterpene hydrocarbons, 4 monoterpene cyclic ethers, 4 furans and 2 aromatic hydrocarbons. It is important to note that although more than 300 VOCs have been reported in other *Citrus* juice [26], some of them have been identified only tentatively [16], [18], [19], [24]. To unequivocally assign chemical names to the compounds in our dataset, we have used analytical grade commercial compounds. Those compounds that were putatively identified by their mass spectra but were not confirmed with the commercial standard were not included in our dataset.

**Figure 1:**
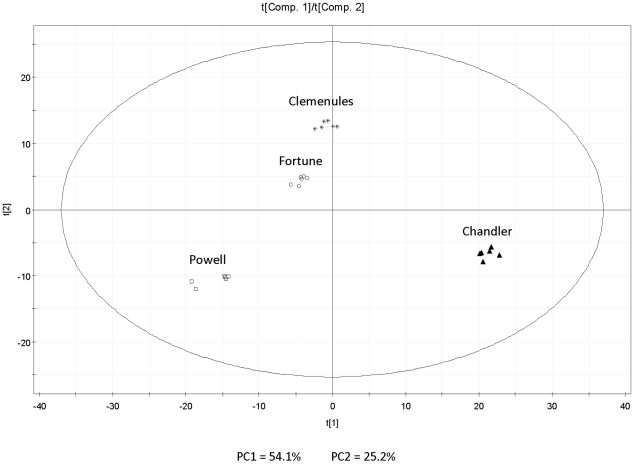
Principal Component Analysis score plot (t[1] vs t[2]) for the first and second principal components.

As a result eleven compounds out of a total of 109 are described here for the first time in the juice of *Citrus* species (6 esters, 2 ketones, 1 furan and 2 aromatic hydrocarbons) (Table 1); the remaining compounds have been described previously in *Citrus* juice samples [11], [16], [17], [24], [26], [32]–[34]. Almost all the detected compounds showed dramatic changes in the levels of accumulation in at least one of the four varieties (see Table 1). To better understand the usefulness of the volatile profile to define and distinguish the four *Citrus* varieties, a principal component analysis (PCA) was performed. Figure 1 shows that the first two principal components explain almost 80% of the variance, and clearly separate all four varieties from one another. The first component, explaining 54% of the variance, mainly separates Chandler pummelo from all the other varieties and to a lesser extent also Powell orange from both Clemenules and Fortune. The second component explains about 25% of the variance and clearly separates Clemenules from Powell and Chandler, while Fortune would be intermediate. Finally, the third component (Figure S1) essentially separates Fortune from the rest, and the analysis of the loading plots should reveal the part of the volatile profile which is characteristic of Fortune, and is responsible of roughly 13% of the total variance. These three components together explain as much as 92% of the total variance in the dataset.

**Figure 2:**
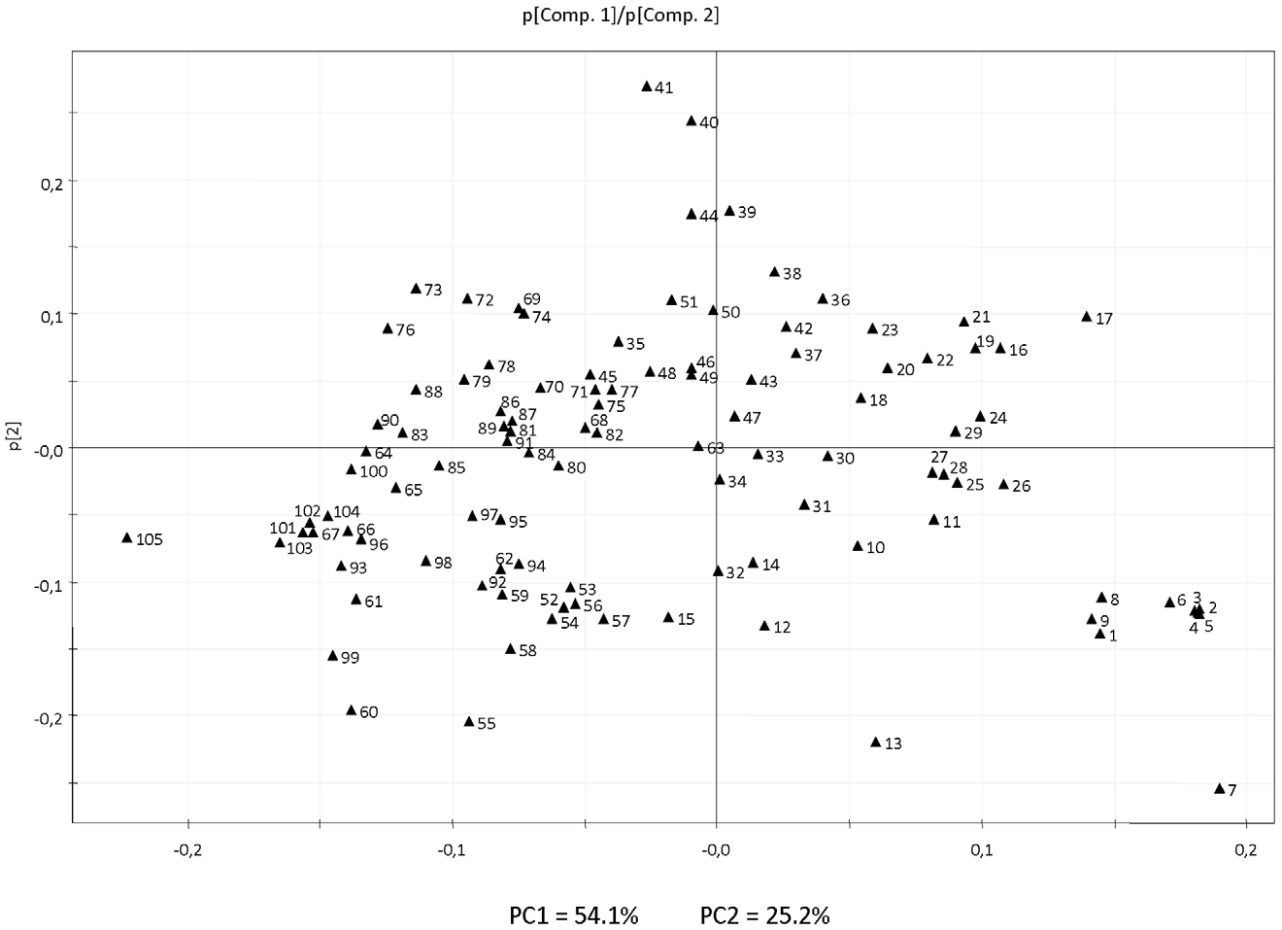
Principal Component Analysis loading plot (p[1] vs p[2]) for the first and second principal components. Each number corresponds to a particular volatile compound, as indicated in Table 1.

Analysis of the loadings plot reveals the compounds responsible of the separation between samples (Figure 2). The most relevant for the first component is a group of mostly terpenic compounds (β-caryophyllene, *(Z*)-ocimene, *(E,E)*-2,4-nonadienal, *(Z)*- and *(E)*-linalool oxides, *p*-cymene) which is almost exclusive of Chandler pummelo, and the compound octyl acetate, a metabolite present at relatively very high levels in Powell. The second component is defined by a group of compounds, mostly esters, with contrasting relative levels between Clemenules and Powell. The most relevant compounds contributing to the separation of Fortune from the other varieties are revealed by the loadings plot corresponding to the third component (Figure S2), and include propyl acetate, citronellyl acetate and ethyl acetate with higher levels in Fortune, and *(E)*-2-hexen-1-ol, eucalyptol, 3-carene and 1-decanol with lower levels in this variety.

**Figure 3:**
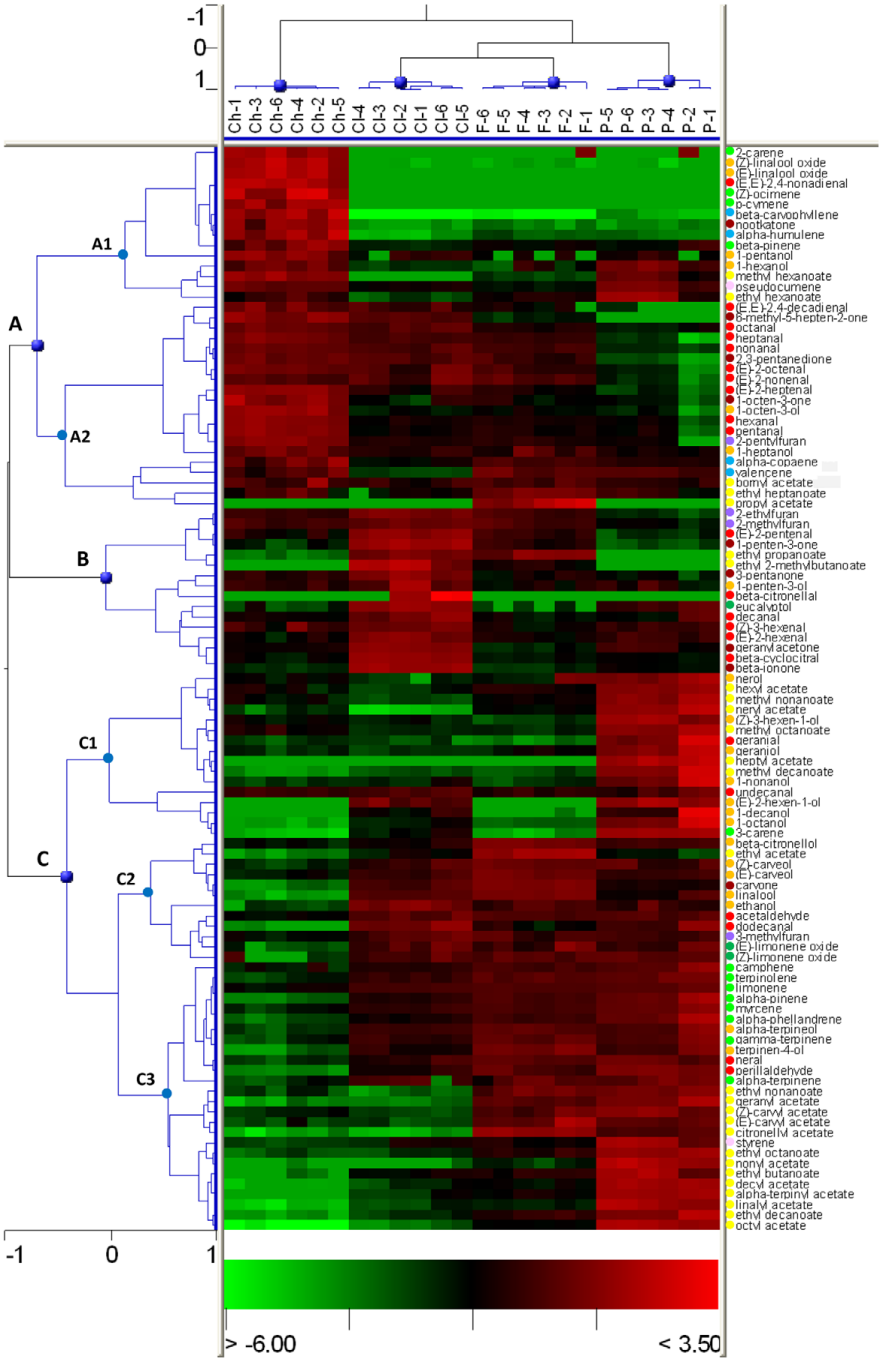
Hierarchical cluster analysis of both samples and identified volatile compounds. Samples grouped themselves by varieties: Ch, Chandler; Cl, Clemenules; F, Fortune; P, Powell. Volatiles grouped in clusters A, B and C, and sub-clusters A1, A2, C1, C2 and C3. Colours in the heatmap mean the fold change, in accordance to the scale in the bottom: red for higher levels; green for lower levels. Colour circles before the name of the compounds describe the chemical family each particular compound belongs to: red, aldehyde; brown, ketone; orange, alcohol; yellow, ester; indigo, furan; pink, aromatic hydrocarbon; light green, monoterpene hydrocarbon; dark green, monoterpene cyclic ether; blue, sesquiterpene.

A hierarchical cluster analysis confirmed that Clemenules and Fortune presented the most similar volatile profile, while Chandler pummelo exhibited the most differential profile of them all (Figure 3). According to the pattern of VOCs presented by these four varieties, volatile compounds can be organized in three clusters, named A, B and C, with some sub-clusters (named A1, A2, C1, C2 and C3). It is therefore revealed that clusters of VOCs with differential accumulation levels rather than a few individual compounds are responsible for the separation between varieties. For the sake of clarity, compounds in Table 1 are displayed according to the same order than in the hierarchical cluster.

**Figure 4:**
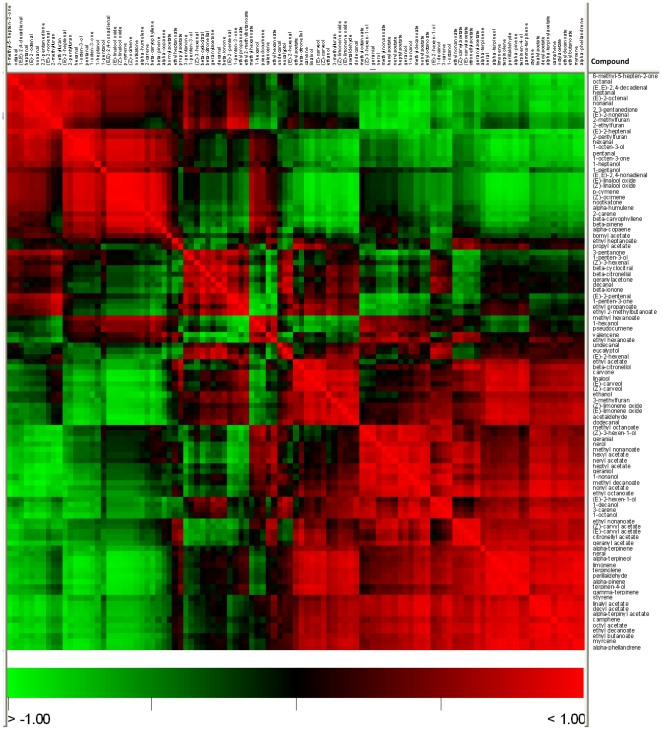
Heatmap of the correlation matrix of the volatile compounds. Positive correlations are shown in red; negative correlations in green; absence of correlation in black.

Correlation analysis of the volatile compounds was also performed, in order to assess how these metabolites were related to each other. When compared to the hierarchical cluster analysis, results are basically consistent. Basically, highly positively correlated volatiles were grouped in the same cluster, and compounds in distant clusters tend to show negative or non-significant correlations (Figure 4, Table S1). When descending to the metabolite to metabolite level, it can be observed a general pattern of high positive correlations of ester compounds to both their alcoholic precursor and other structurally similar esters. This suggests that the levels of these compounds, which show up to 500-fold variations between varieties, could be regulated both by enzymatic activity (by means of relatively specific alcohol acyl transferases) and by substrate availability. A strong negative correlation between ester and aldehyde levels is also observed. This also suggests an important role for alcohol dehydrogenase enzymes activity in the differences detected between the volatile profiles of Chandler, otherwise basically rich in sesquiterpenes and aliphatic aldehydes, and the other varieties with a volatile profile with higher abundance of alcohols and esters.

Compounds in the cluster A are present at higher levels in Chandler pummelo than in any of the other three varieties studied. Compounds which are basically exclusive of Chandler belong to sub-cluster A1 and include mostly monoterpene hydrocarbons and derivatives such as 2-carene, (Z)-linalool oxide, (E)-linalool oxide, (Z)-ocimene, p-cymene, and also (E,E)-2,4-nonadienal and nootkatone. Among the compounds in sub-cluster A1, 2-carene had only been identified so far in pummelo peel oil [35] and the sesquiterpene nootkatone has been frequently described in grapefruit juice [4] but rarely in other Citrus juices [18]; the remaining compounds in this subcluster have been identified also in Citrus juices [3], [17], [26]. Sub-cluster A2 includes aliphatic aldehydes from five to nine carbon atoms, and some olefinic aldehydes such as (E)-2-heptenal, (E)-2-octenal, (E)-2-nonenal, and (E,E)-2,4-decadienal, all of which have been described to provide herbal, fruity and floral aroma to Citrus juices [26], [32]. This sub-cluster also includes the compound 2-pentylfuran, reported previously only in tangerine [34], but identified in all four of our varieties in this paper. Cluster A included the only four sesquiterpenes unambiguously identified in our analysis: β-caryophyllene, nootkatone, α-copaene and valencene (β-farnesene was only detected at the level of traces in Powell), all of which had been previously reported in Citrus juices [17], [26]. However, the chromatograms of all varieties, and most notably those of Chandler, presented a large number of unidentified sesquiterpenes (as could be inferred from their MS spectra) which corresponded to the most abundant peaks eluting between 35 and 41 min (Figure S3). The close similarity of the mass spectra of many sesquiterpenes and the lack of standards makes this identification difficult, as it requires the use of purification steps and additional analytical techniques (such as NMR, and chemical synthesis) in order to identify their exact molecular structures. Therefore, although noted here, we did not include them in our approach.

Cluster B is defined by the compounds more abundantly found in Clemenules than in any of the other three varieties. These include a set of highly correlated carotenoid derivatives probably by the action of carotenoid cleavage dioxygenases: β-cyclocitral, β-ionone and geranylacetone (Figure 4, Table S1), and 3-pentanone, a ketone reported here for the first time in a *Citrus* juice. Other compounds in cluster B have also been previously described in *Citrus* juice [34] and they include 1-penten-3-one, 2-ethylfuran, 2-methylfuran, eucalyptol and the aldehydes *(E)*-2-pentenal, decanal, *(Z)*-3-hexenal, *(E)*-2-hexenal, and finally β-citronellal, which in our analysis was only detected in Clemenules.

Sub-cluster C1 includes compounds found more abundant in Powell than in the other three varieties. The monoterpene 3-carene and the esters methyl octanoate, methyl decanoate and heptyl acetate are the most important (heptyl acetate is exclusive of Powell variety). Methyl octanoate and methyl decanoate had never been described in *Citrus* juice, although the presence of many other esters had been previously reported in *Citrus* [16], [17], [26]. Sub-cluster C2 includes most of the compounds which accumulated generally to higher levels in Fortune than in other varieties, such as linalool or β-citronellol.

Finally, sub-cluster C3 includes compounds which are present in smaller quantities in Chandler than in the other varieties studied. Included in this sub-cluster are monoterpene hydrocarbons such as α-phellandrene, limonene or γ-terpinene, all of which are generally described in *Citrus* juices [26]. Also neral and perillaldehyde aldehydes, and 3-methylfuran (the only one of the four furans detected here that had never been described in *Citrus* juice before) were less abundant in Chandler than in the other three varieties. Some furan compounds are considered to be originated from lipid oxidation [36], but our results suggest independent metabolic pathways for the synthesis of 2- and 3-alkyl furans. This is based in 2-methylfuran showing a very strong positive correlation to 2-ethylfuran and also to 2-pentylfuran in our samples, while no significant correlation was found to 3-methylfuran. Moreover, the majority of compounds included in this sub-cluster showed the highest levels in Powell variety, as it is the case for monoterpenes limonene, α-phellandrene and α-pinene, monoterpene acetates, aliphatic esters octyl-, nonyl-, and decyl acetate, ethyl octanoate, ethyl nonanoate and ethyl decanoate (ethyl nonanoate never been described in *Citrus* literature before) and the aromatic hydrocarbon styrene. Styrene and pseudocumene (other aromatic hydrocarbon synonymous of 1,2,4-trimethylbenzene) have been identified in all our four varieties for the first time. Of these two, only styrene have been described previously in *Citrus* commercial juices [21] but not pseudocumene although a compound with a similar structure, 1,4-diethylbenzene, have been reported previously in tangerine juice [37].

Some volatile compounds commonly described in *Citrus* juices failed to be detected in our study (Table S2). Thus, no volatile acids were detected in the juices analyzed; in fact it is known that the contribution of the acids to the total aroma of the orange juice is very limited [24]. In addition, some esters usually described in the *Citrus* juice, such as methyl butanoate [3], [14], [15], [28], ethyl 3-hydroxyhexanoate [5], [16], [28], [33], or methyl *o*-(methylamino)benzoate [17] were not identified in our samples. Moreover some alcohols such as the aliphatic alcohols 2- and 3-methylbutanol [3] or the monoterpene alcohol borneol and sesquiterpene alcohols β-eudesmol and α-bisabolol [12] described in previous *Citrus* analysis were not found in our samples. Vanillin was not found in our samples either, although it has been described in many other studies in *Citrus* juices [5], [27], although this compound usually appears in juices that have undergone degradation due to exposure to high temperature [26]. This is also the case for some aldehydes identified in *Citrus* aroma, such as cuminaldehyde o *(E)*-2-undecenal [4], [13], or some C_13_-norisoprenoids such as β-damascenone or α-ionone identified previously in orange juice [24]. Overall lack of detection of some of those compounds in our samples could be due to these compounds not being present in our samples because of biological/environmental variability, although we cannot discard that differences in extraction and analytical techniques used (i.e. exposure of juices to high temperatures) or misidentification of those compounds in previous reports could be the reason.

In summary, over 100 volatile compounds have been unequivocally identified for the first time in the juice of four varieties of *Citrus* using the same analytical conditions, and therefore allowing us to perform more robust comparisons. Cluster and correlation analyses indicated interesting relationships between compounds and classes of compounds revealing the existence of interesting interactions between the biosynthetic pathways. Our results revealed also that the differences in the volatile profile in *Citrus* juice are mainly quantitative, and only a few compounds are variety-specific. What appears to be specific is the profile, i.e. relative content of a set of volatiles. Thus, according to the volatile profile, the most different varieties were Chandler and Powell, while Clemenules and Fortune were intermediate and very similar to one another. In Chandler the most characteristic volatiles were principally aliphatic aldehydes, sesquiterpenes such as nootkatone and monoterpenes such as 2-carene. Powell Navel orange showed the highest levels of esters such as nonyl acetate and of monoterpenes such as 3-carene. Clemenules showed the highest levels of ketones 3-pentanone and β-ionone and Fortune showed the highest levels of some acetate esters such as ethyl and propyl acetate, this latter almost Fortune-exclusive.

Volatile profiling of Citrus juice by HS-SPME-GC-MS has proven therefore to be a highly valuable tool for the characterization of fruit from different varieties. The results and volatile platform described in this paper could be used as a roadmap to guide in the selection process of *Citrus* breeding programs directed to obtain new varieties with better aroma, to monitor industrial processes that may affect aroma, and also in the study of the pathways leading to volatile production in *Citrus.*

## Supporting Information

Figure S1 **Principal Component Analysis score plot (t[1]** **vs t[3]) for the first and third principal components.**
(TIF)

Figure S2 **Principal Component Analysis loading plot (p[1]** **vs p[3]) for the first and third principal components.** Each number corresponds to a particular volatile compound, as indicated in Table 1.
(TIF)

Figure S3 **Chromatograms representing each of the varieties analyzed: A, Chandler; B, Clemenules; C, Fortune; D, Powell.**
(TIF)

Table S1 **Pearson correlation coefficients for each of the volatile compounds.** Significant correlations (p<0.01) are highlighted in bold.
(XLS)

Table S2 **Volatile organic compounds injected as standard but not identified in any juice analyzed.**
(DOC)
