# Supplementary figures and images for: Comparative Analysis of the Volatile Fraction of Fruit Juice from Different Citrus Species
Source: PLoS One. 2011 Jul 19;6(7):e22016. doi: 10.1371/journal.pone.0022016 (PMC3139606; doi:10.1371/journal.pone.0022016)

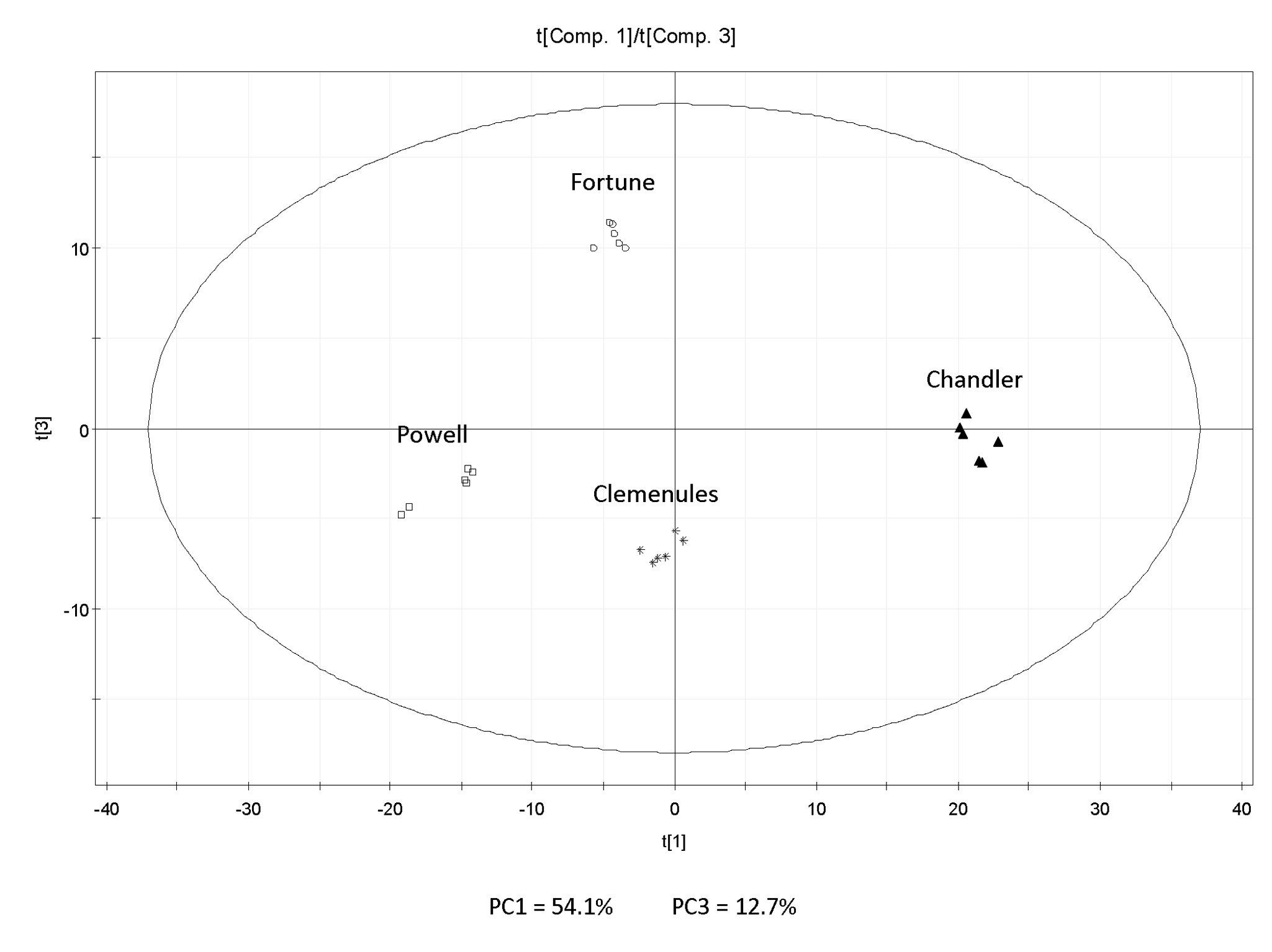

Supplement: Figure S1 — Principal Component Analysis score plot (t[1] vs t[3]) for the first and third principal components. (TIF) [file pone.0022016.s001.tif]

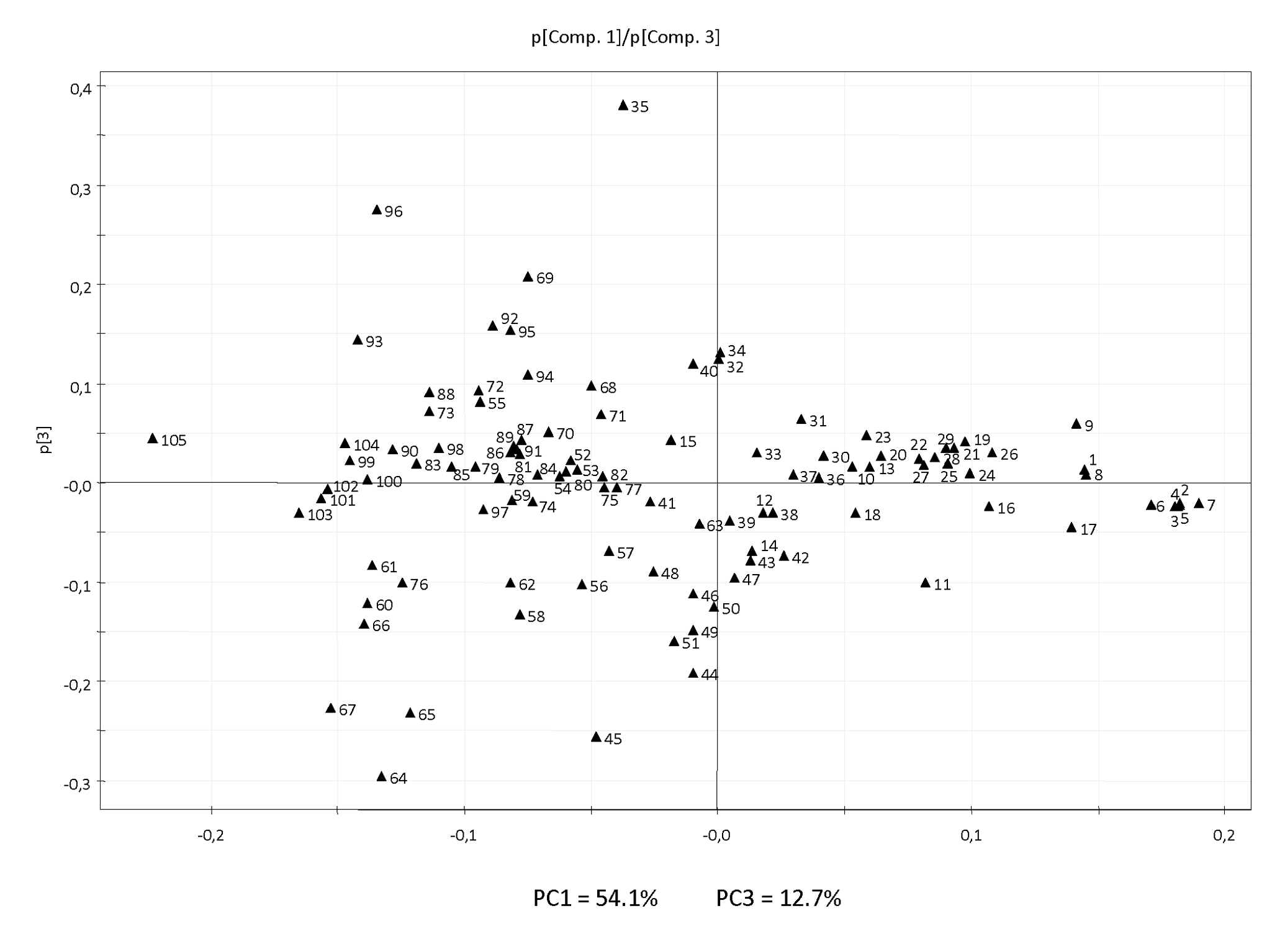

Supplement: Figure S2 — Principal Component Analysis loading plot (p[1] vs p[3]) for the first and third principal components. Each number corresponds to a particular volatile compound, as indicated in Table 1. (TIF) [file pone.0022016.s002.tif]

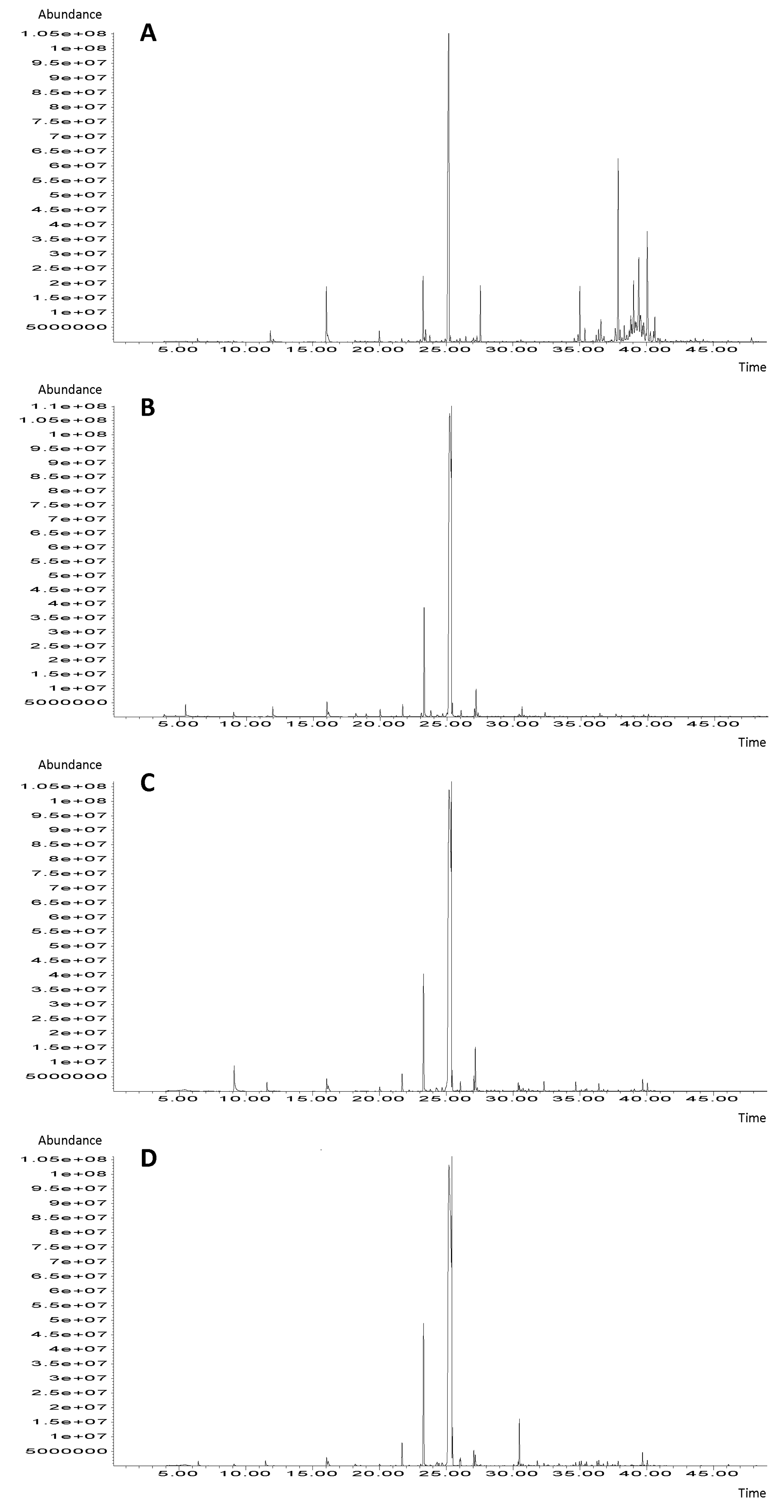

Supplement: Figure S3 — Chromatograms representing each of the varieties analyzed: A, Chandler; B, Clemenules; C, Fortune; D, Powell. (TIF) [file pone.0022016.s003.tif]
