# Supplementary material for: Comparative Analysis of the Volatile Fraction of Fruit Juice from Different Citrus Species
Source: PLoS One. 2011 Jul 19;6(7):e22016. doi: 10.1371/journal.pone.0022016 (PMC3139606; doi:10.1371/journal.pone.0022016)
Supplement: Table S2 — Volatile organic compounds injected as standard but not identified in any juice analyzed. (DOC) [file pone.0022016.s005.doc]

Table S2. Volatile organic compounds injected as standard but not identified in any juice analyzed.

| Compound | | Retention time (min) |
| --- | --- | --- |
| *Acids* | | |
| **1** | Butanoic acid | 14.43 |
| **2** | 3-methylbutanoic acid | 16.77 |
| **3** | Pentanoic acid | 18.31 |
| **4** | Hexanoic acid | 22.06 |
| **5** | 2-ethylhexanoic acid | 27.03 |
| **6** | Octanoic acid | 28.78 |
| *Aldehydes* | | |
| **7** | Ethanal | 4.98 |
| **8** | *(E,E)*-2,4-hexadienal | 20.43 |
| **9** | Phenylacetaldehyde | 25.62 |
| **10** | Salicylaldehyde | 25.71 |
| **11** | *p*-tolualdehyde | 27.17 |
| **12** | Myrtenal^a^ | 31.03 |
| **13** | Cuminaldehyde | 32.35 |
| **14** | Cinnamaldehyde | 33.30 |
| **15** | 10-undecenal | 33.48 |
| **16** | *(E)*-2-undecenal | 35.38 |
| *Alcohols* | | |
| **17** | 3-methylbutanol | 13.41 |
| **18** | 2-methylbutanol | 13.55 |
| **19** | 2,3-butanediol | 15.15 |
| **20** | 4-methyl-1-pentanol | 17.38 |
| **21** | *(E)*-3-hexen-1-ol | 17.92 |
| **22** | *(Z)*-4-hepten-1-ol | 22.26 |
| **23** | 6-methyl-5-hepten-2-ol | 23.20 |
| **24** | *(Z)*-3-octen-1-ol | 25.61 |
| **25** | *(E)*-2-octen-1-ol | 25.93 |
| **26** | Guaiacol | 26.99 |
| **27** | 2-nonanol | 27.05 |
| **28** | 2-phenylethanol | 27.95 |
| **29** | Fenchyl-alcohol^a^ | 28.53 |
| **30** | *(E)*-2-nonen-1-ol | 29.30 |
| **31** | Isoborneol^a^ | 29.98 |
| **32** | Menthol^a^ | 30.16 |
| **33** | Borneol^a^ | 30.28 |
| **34** | Thymol^b^ | 33.20 |
| **35** | Perillyl alcohol^a^ | 34.00 |
| **36** | 10-undecen-1-ol | 35.23 |
| **37** | Eugenol | 35.37 |
| **38** | Undecanol | 35.62 |
| **39** | Vanillin | 36.73 |
| **40** | 1-dodecanol | 38.21 |
| **41** | *(Z)*-nerolidol^c^ | 39.85 |
| **42** | *(E)*-nerolidol^c^ | 40.64 |
| **43** | Cedrol^c^ | 43.21 |
| **44** | β-eudesmol^c^ | 43.93 |
| **45** | α-bisabolol^c^ | 44.03 |
| *Esters* | | |
| **46** | Methyl butanoate | 12.92 |
| **47** | Butyl acetate | 16.39 |
| **48** | Methyl pentanoate | 16.80 |
| **49** | Propyl butanoate | 19.66 |
| **50** | Ethyl pentanoate | 19.68 |
| **51** | Pentyl acetate | 20.21 |
| **52** | (Z)-3-hexenyl acetate | 23.67 |
| **53** | (E)-2-hexenyl acetate | 24.95 |
| **54** | 2-pentyl butanoate | 24.25 |
| **55** | Methyl heptanoate | 24.31 |
| **56** | Methyl-3-hydroxyhexanoate | 25.50 |
| **57** | Pentyl butanoate | 26.71 |
| **58** | Ethyl-3-hydroxyhexanoate | 27.99 |
| **59** | Diethyl butanedioate  (Diethyl succinate) | 29.36 |
| **60** | Hexyl butanoate | 29.96 |
| **61** | Methyl salicylate | 30.80 |
| **62** | Heptyl butanoate | 32.95 |
| **63** | Methyl *o*-(methylamino) benzoate  (Methyl-N-methylanthranilate) | 37.10 |
| **64** | Ethyl-2-aminobenzoate  (ethyl anthranilate) | 37.28 |
| **65** | Undecyl acetate | 38.97 |
| **66** | Dodecyl acetate | 41.35 |
| *Ketones* | | |
| **67** | 2,3-butanedione | 8.42 |
| **68** | 3-buten-2-one  (methyl vinyl ketone) | 8.47 |
| **69** | 2-butanone | 8.72 |
| **70** | 4-pentalactone | 22.06 |
| **71** | Acetophenone | 24.46 |
| **72** | γ-hexalactone | 25.66 |
| **73** | Sotolon | 27.05 |
| **74** | Fenchone (R) (-)^a^ | 27.42 |
| **75** | α-thujone^a^ | 27.92 |
| **76** | γ-heptalactone | 29.12 |
| **77** | γ-octalactone | 32.43 |
| **78** | β-damascenone^d^ | 36.21 |
| **79** | α-ionone^d^ | 37.40 |
| **80** | Dihydro-β-ionone^d^ | 37.74 |
| **81** | γ-decalactone | 38.43 |
| **82** | γ-undecalactone | 41.16 |
| **83** | δ-dodecalactone | 44.48 |
| *Sesquiterpene hydrocarbons* | | |
| **84** | α-longipinene | 35.90 |
| **85** | Sativene | 37.08 |
| **86** | α-cedrene | 37.91 |
| **87** | β-humulene | 38.78 |
| **88** | Ledene | 39.62 |
| *Others* | | |
| **89** | 1-dodecyne | 30.97 |
| **90** | Anethole | 33.57 |
| **91** | Azulene | 35.01 |

^a^Monoterpene derived compound. ^b^Monoterpenic phenol. ^c^Sesquiterpene derived compound. ^d^Norcarotenoid (C_13_-Norisoprenoid) compound.
